# Electrocatalytic Anaerobic Oxidation of Benzylic Amines Enabled by Ferrocene-Based Redox Mediators

**DOI:** 10.1021/acs.organomet.4c00219

**Published:** 2024-08-11

**Authors:** Amy L. Waldbusser, Shabnam Hematian

**Affiliations:** Department of Chemistry and Biochemistry, University of North Carolina at Greensboro, Greensboro, North Carolina 27402, United States

## Abstract

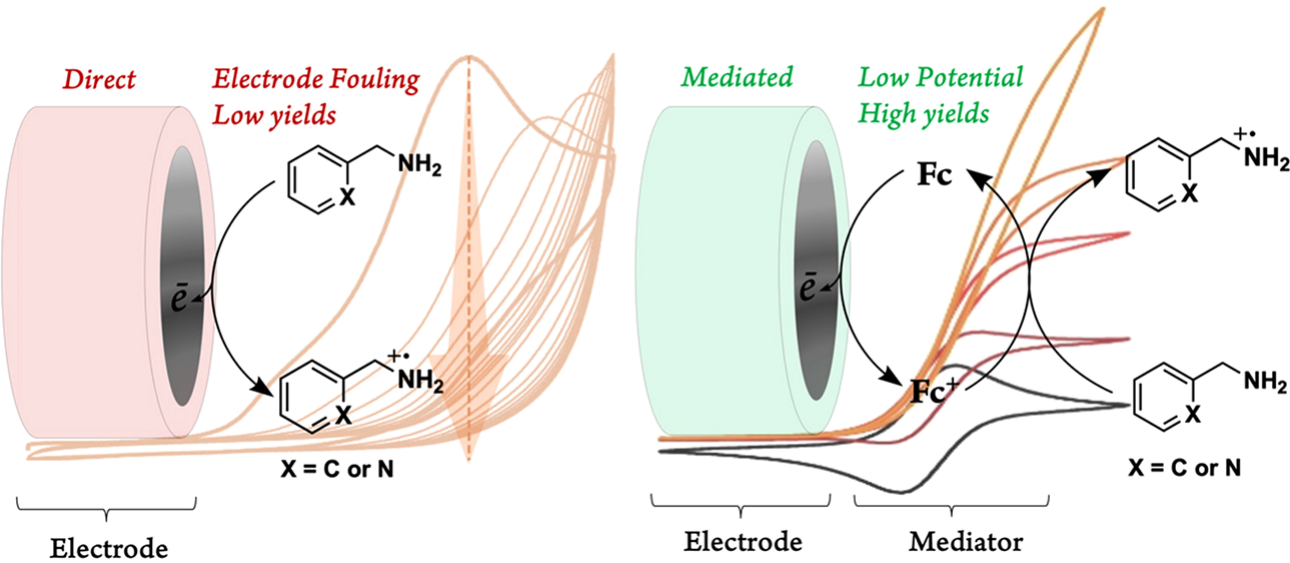

The generation and functionalization of carbon- or nitrogen-centered radicals are of great interest for their potential synthetic utility. Here, we report the anaerobic electrocatalytic oxidation of two primary benzylic amines, benzylamine and 2-picolylamine, in the presence of a catalytic quantity of an electron deficient ferrocene derivative as a single-electron redox mediator. The use of the appropriate redox mediator prevented fouling of the electrode surface and significantly decreased the potential at which the catalytic oxidation reaction occurred. Simulation of the electrochemical results revealed an E_r_C_i_′ catalytic process between the redox mediator and both substrates and significant difference in the electron transfer rate between the two substrates and electrochemically oxidized mediator. Through anaerobic controlled-potential electrolysis, we demonstrated a method with a Faradaic efficiency of 90% forming the desired coupled imine product of benzylamine oxidation while avoiding an excess of problematic overoxidation, hydrolysis, and other side reactions. Based on the electrochemical data along with the product analyses using IR and ^1^H and ^13^C NMR spectroscopies, the proposed mechanistic steps for the redox mediated electrocatalytic process were laid out.

## Introduction

Carbon- or nitrogen-centered radical species are attractive intermediates involved in a variety of chemical transformations forming C–C and C–N bonds. They can be generated through numerous synthetic methods to functionalize molecules in organic synthesis and convert simple compounds into chemicals of higher complexity and value.^1^ Particularly, due to the ubiquity of nitrogen-containing functionalities in specialty and commodity chemicals, the ability to controllably oxidize these substrates to access radical and electrophilic groups is an important synthetic goal. However, conventional methods for these types of reactions typically involve undesirable reaction conditions, including high temperatures, large amounts of harsh oxidants, and expensive catalysts.^1^ Electrochemistry can offer a mild and versatile alternative and provides a powerful approach to access the radical intermediates due to its precise control over redox processes.^2^ For this reason, electrochemistry has become more recognized as a powerful tool to develop new synthetic methods for sustainable chemical reactions over recent years.^3−5^ There are examples of electrochemical oxidation involving one-electron pathways to selectively form and functionalize a radical species. However, the radical species most often reacts with dioxygen present to form the oxygenated product,^6−8^ and there are limited reports on anaerobic functionalization of the radical.^9,10^ Additionally, avoiding expensive electrode materials such as the use of carbon-based electrodes is preferable from a cost point of view.^11^

The electrochemical oxidation of amines, particularly primary amines, presents its own challenges, most notably the reactivity of the singly oxidized species (i.e., radicals) toward the carbon-based working electrode, causing surface fouling which shuts down the flow of electrons between the reaction solution and the electrode surface.^12^ Additionally, primary amines are more difficult to oxidize than secondary or tertiary amines, and require higher overpotentials for oxidation, which can result in a greater chance of over oxidizing the amines and lead to substrate degradation or electrode surface fouling.

One way to overcome these challenges is through the use of a redox mediator, a compound that is continuously oxidized at the electrode surface and in turn reduced by the substrates in solution. This keeps the oxidized substrate away from the electrode surface, preventing passivation of the electrode and encouraging the desired reactions to occur instead. Effective mediators undergo reversible redox processes, at potentials less positive than that of the substrate, allowing the target oxidation reaction to occur at lower potentials than would be required without a mediator and thus preventing over oxidation. Redox mediators should include appropriate reduction potentials, high stability of both oxidized and reduced forms in the reaction conditions, and fast electron-transfer kinetics.

Ferrocene (Fc) is an attractive compound for this purpose, due to its well-known reversible one-electron redox process between the ferrocene and ferricenium (Fc^+^) states.^13,14^ In fact, there have been recent reports documenting the efficacy of ferrocene as a redox mediator for the electrocatalytic oxidation of various organic substrates, including amines and amides.^10,13,15−19^ However, there have not been such studies performed with benzylic amines. Yet, the oxidation of benzylamines through various methods, ranging from thermally assisted chemical^20−28^ to photochemical oxidation reactions,^29−32^ is known to result in the formation of several byproducts including the respective secondary imines. Deb et al. also demonstrated that this reaction could be carried out under mild conditions using a ferricenium catalyst in water and air as the primary oxidant.^33^ Many of the hypothesized mechanisms for benzylamine oxidation not only require the use of dioxygen (O_2_) as the terminal oxidant to drive the reaction forward but also depict the role of O_2_ as necessary in many catalytic steps involving formation of the hypothetical superoxide, hydroperoxide, or other reactive oxygen species, which can lead to undesired products. Another common side reaction, due to the presence of water, is hydrolysis of the oxidized products.^20,29,31^ Ultimately, however, it would be desirable to control the reactivity through anaerobic methods, such as redox mediated electrocatalytic oxidation that can form and functionalize the radical species and selectively result in the desired product.

The results herein demonstrate the effectiveness of electron-deficient ferrocene derivatives to act as redox mediators in the anaerobic electrocatalytic oxidation of benzylic amines to selectively form coupled imine products. Cyclic voltammetry studies reflect the catalytic nature of the reaction conditions through an E_r_C_i_’ mechanism lowering the potential required for amine oxidation. Through controlled potential electrolysis, the desired product was formed with no evidence of hydrolysis, giving a new route to performing efficient amine oxidations that can be an impactful strategy for the future of electrochemical organic synthesis.

## Results and Discussion

### Cyclic Voltammetry

**Figure 1:**
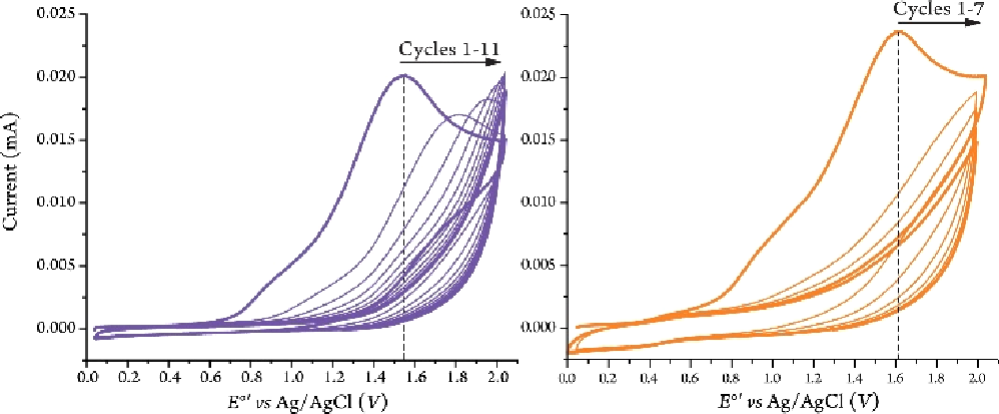
Cyclic voltammograms of the direct oxidation of 1 mM benzylamine (left) and 2-picolylamine (right) at 100 mV s^–1^, started at 0.0 V, in MeCN with 100 mM [(*n*Bu)_4_N][PF_6_] as the supporting electrolyte, exhibiting passivation of the glassy carbon electrode as cycles continued.

The cyclic voltammogram of the direct oxidation of benzylamine (BA) in an acetonitrile (MeCN) solution with 100 mM of [(*n*Bu)_4_N][PF_6_] as the supporting electrolyte revealed an irreversible oxidation peak at 1.55 V vs Ag/AgCl that passivated the surface of the working electrode over several cycles, as was evident by the loss of a peak shape and a decrease in current as the cycling continued (Figure 1, left). A similar surface fouling behavior was also observed for 2-picolylamine (PA), which displayed an irreversible peak at 1.62 V vs Ag/AgCl (Figure 1, right).

**Scheme 1:**
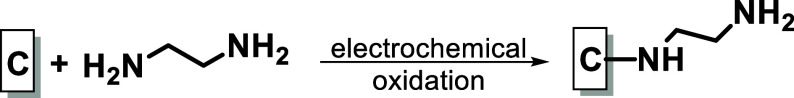
Attachment of Oxidized Primary Amine to a Carbon Electrode Surface Recreated with permission from ref (34). Copyright 1990 IOP Publishing.

This was expected, as primary amines are known to adhere to the surface of carbon electrodes upon electrochemical oxidation, specifically through the nitrogen radical cation intermediate that initially forms, as proposed first by Desarmot and Sanchez in 1990 (Scheme 1).^34^ The maximum current observed in the first scans is similar for both amines, pointing to the comparable diffusion rates of the substrates in our experimental condition (Figure 1).

**Figure 2:**
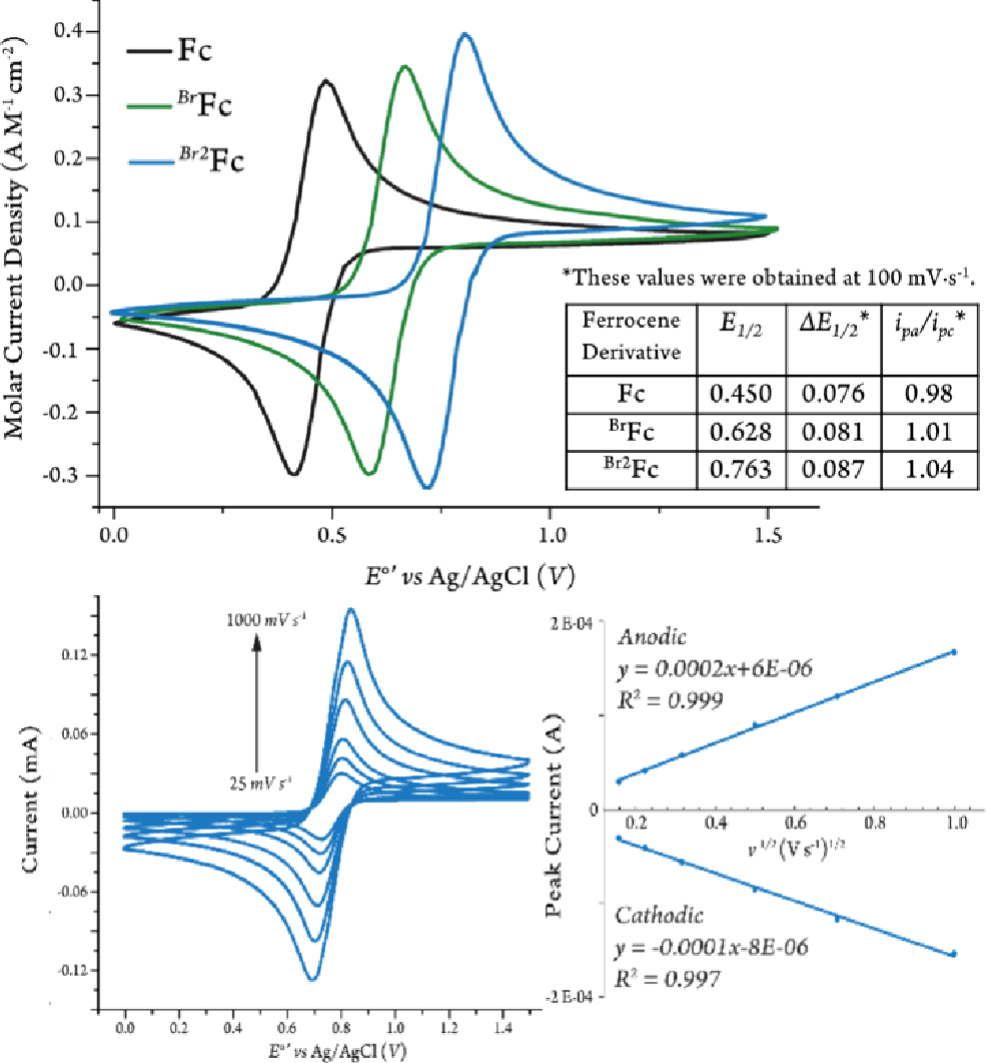
Cyclic voltammograms and related values (*top*) for the ferrocene derivatives at 100 mV s^–1^, started at 0.0 V, in MeCN with [(*n*Bu)_4_N][PF_6_] as the supporting electrolyte (100 mM). Randles-Sevcik plot for ^*Br2*^Fc (2 mM) (*bottom*) serving as a reprehensive example for the diffusion measurements.

In order to overcome electrode surface fouling, we employ ferrocene derivatives as single-electron electrochemical mediators with an appropriate range of reduction potentials. As part of a larger study conducted within our laboratory,^35^ many ferrocene derivatives were examined through cyclic voltammetry in various solvent/electrolyte conditions for the reduction potential, reversibility, stability, and diffusion rates.^36^ Two electron-deficient ferrocene derivatives [i.e., bromoferrocene (^*Br*^Fc) and *1,1’*-dibromoferrocene (^*Br2*^Fc)] as well as the parent ferrocene complex were selected for this study due to their superior electrochemical performance.^35^ In an acetonitrile (MeCN) solution containing 100 mM [(*n*Bu)_4_N][PF_6_] as the supporting electrolyte, the introduction of an electron-withdrawing bromo-substituent on one or both cyclopentadienyl ring(s) increases the reduction potential by 178 mV or 313 mV (Figure 2, top). Their redox processes were quite reversible (i.e., *ΔE*_1/2_ ranging from 76 to 87 mV, anodic/cathodic peak current ratios (i_pa_/i_pc_) between 0.98 and 1.04), and all three complexes in both reduced and oxidized forms were freely diffusing through the solution, confirming that these derivatives can act as appropriate redox mediators for an electrocatalytic oxidation process.

Among the derivatives studied, *^Br2^*Fc displayed the most suitable reduction potential value for the oxidation of benzylic amines. The one-electron redox process for ^*Br2*^Fc^+^/^*Br2*^Fc at 0.763 V vs Ag/AgCl was reversible, as shown in Figure 2. Interestingly, a Randles-Sevcik analysis of the peak current vs the square root of the scan rate revealed that ^*Br2*^Fc, despite its higher molecular weight, shows relatively higher diffusion coefficient values for both oxidized and reduced species (1.50 and 1.81 × 10^–7^ cm^2^s^–1^, Figure 2, bottom) when compared to those of other ferrocene derivatives.^35^ These data demonstrate the ability of ^*Br2*^Fc to serve as an effective redox mediator for electrocatalytic oxidation of benzylic amines.

**Figure 3:**
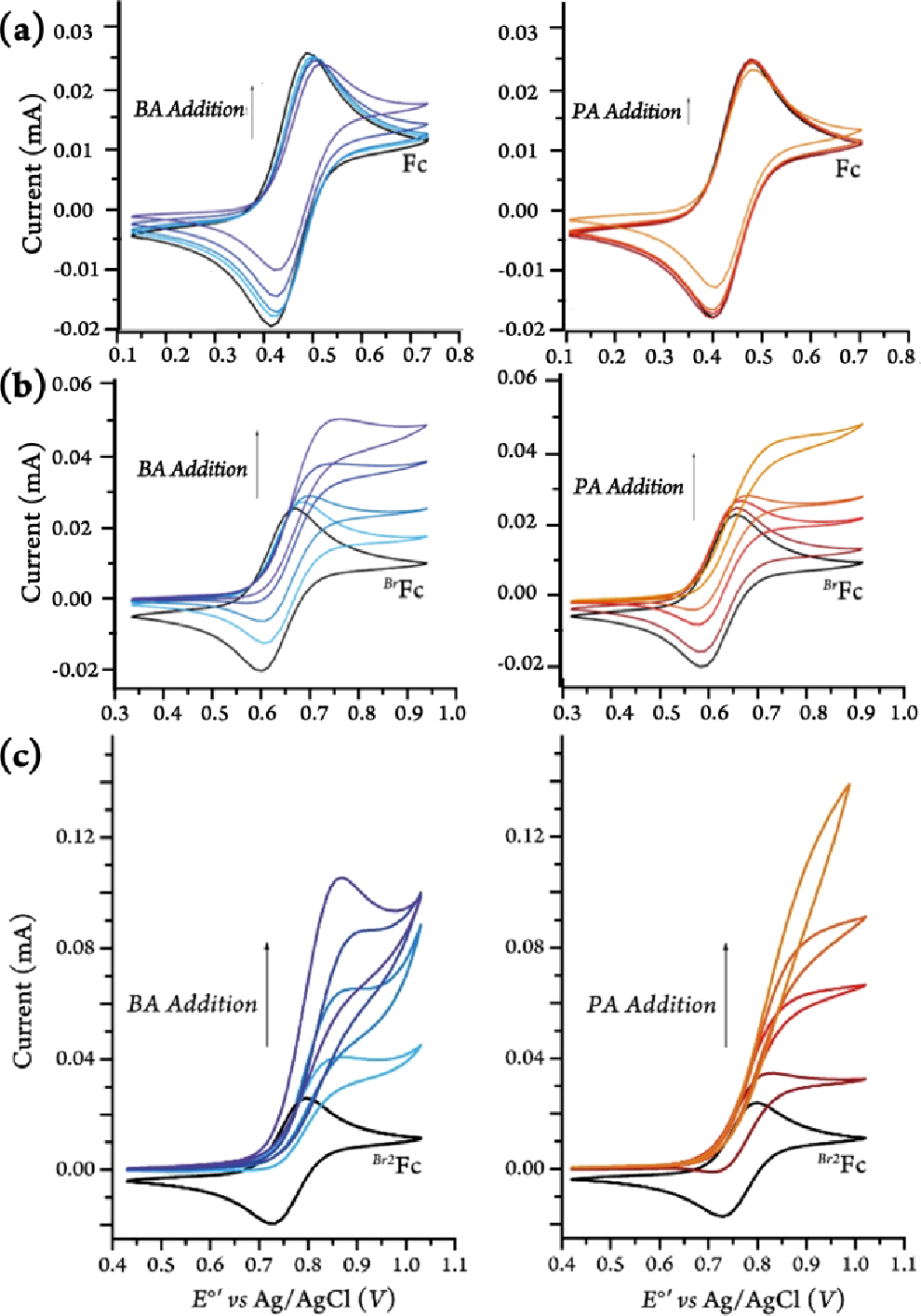
Cyclic voltammograms of 1 mM (a) Fc, (b) ^*Br*^Fc, or (c) ^*Br2*^Fc in MeCN (100 mM [(*n*Bu)_4_N][PF_6_]) (black) with the addition of 100, 250, 500, and 1000 mM of BA (*left*) and 10, 50, 100, and 500 mM of PA (*right*) at 100 mV s^–1^. The scans were started at (a) 0.13, (b) 0.33, (c) 0.43 V, respectively.

Cyclic voltammetry measurements of the three chosen ferrocene derivatives, Fc, ^*Br*^Fc, and ^*Br2*^Fc, in the presence of increasing concentrations of BA and PA were measured to determine which ferrocene would act as the most effective electrocatalytic mediator. The shape of the CV responses indicate whether or not catalytic activity is occurring, as described by Saveant.^37^ As shown in Figure 3a, ferrocene did not perform as a redox mediator in the presence of either amine, as evident by its voltammogram remaining duck-shaped throughout the measurement. This is most likely due to its much lower reduction potential as compared to the peak oxidation potential values of both BA and PA. ^*Br*^Fc offered a slightly more catalytic response, but only in the highest concentrations of BA and PA at 100 mV s^–1^ (Figure 3b). Figure 3c demonstrates the CV responses of ^*Br2*^Fc obtained with increasing concentrations of BA and PA at 100 mV s^–1^. Notably, electrode surface fouling was no longer observed when ^*Br2*^Fc was present with each amine. Instead, the current increased from the current acquired with ^*Br2*^Fc alone, representing a reversible electron transfer (from the amine to the electrochemically generated ferricenium species) followed by an irreversible homogeneous chemical reaction, also known as an E_r_C_i_′ catalytic mechanism, in which the redox mediator is regenerated on the time scale of the scan rate and is proportional to catalyst activity.^37−39^ Additionally, the loss of the cathodic peak further indicated reactivity. The CV responses for this process depend on the parameters
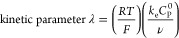

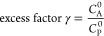
where *k*_e_ is the rate constant of the electron transfer from the electrochemically generated ferricenium to the amine, *C*_*P*_^0^ is the concentration of the redox mediator, *C*_*A*_^0^ is the concentration of the amine, and ν is the scan rate. *R*, *T*, and *F* are the ideal gas constant, temperature of the reaction, and Faraday’s constant.^38^

Other important factors to note are the catalysis-initiating reduction potential (*E*_*redox*_), the potential necessary for catalysis (*E*_*cat*_) and the half-wave potential (*E*_*cat/2*_). *E*_*redox*_ is the reduction potential of the mediator (^*Br2*^Fc) without the amine which provides thermodynamic information about the reaction, and *E*_*cat/2*_ is the potential at which half of the maximum catalytic current is measured and provides kinetic information. Determining E_*cat*_ has not been consistent among reports, as some suggest that this is the potential at which the catalytic peak begins (“onset”), while others use the potential of the peak current. For this reason, it has been suggested by Dempsey and co-workers that the most effective way to study the effect of a redox mediator on catalysis is to look at the *E*_*cat/2*_ value.^38^ For our systems, both *E*_*cat*_ and *E*_*cat/2*_ values are presented in Table S1, where we measured *E*_*cat*_ as the potential at the maximum current. It was found that the E_cat/2_ value for both BA and PA decreases by approximately 430 mV when the amines are oxidized with ^*Br2*^Fc^+^ as a redox mediator as compared to their direct electrochemical oxidations. Additionally, both E_*cat/2*_ values are very close to the *E*_*redox*_ of ^*Br2*^Fc (*E*_*cat/2*_ = 0.798 V, *E*_*redox*_ = 0.763 V), which is expected for an efficient catalytic system.

**Figure 4:**
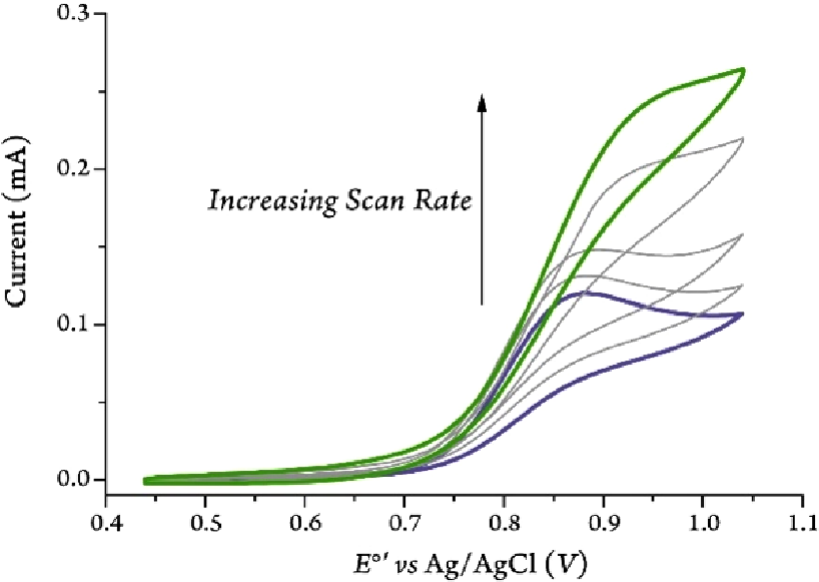
Cyclic voltammograms of ^*Br2*^Fc (1 mM) in MeCN (100 mM [(*n*Bu)_4_N][PF_6_]) in the presence of 1 M benzylamine at scan rates of 100, 250, 500, and 3500 mV s^–1^, started at 0.43 V.

In the case of benzylamine, at a 100 mV s^–1^ scan rate, the voltammogram was the most S-shaped at 100 mM, and then began to peak again at concentrations above 500 mM. This can be ascribed to competition between the substrate oxidation and the diffusion of new substrate toward the electrode.^37,38^ This was mostly avoided by increasing the scan rate, as seen in Figure 4, where the voltammogram became less peak-shaped as the scan rate increased.

With 2-picolylamine, the S-shaped voltammograms appeared at much lower concentrations of the amine (10 mM) and did not form into a peaked-shape response even at a concentration of 500 mM. This demonstrates that the amine stayed at equal concentrations both in the bulk solution and at the electrode. Comparison between CV responses for both substrates in similar conditions (i.e., scan rate, substrate and mediator concentrations, and diffusion rates) implies that the rate of electron-transfer from the substrate to the ferricenium species generated during electrocatalysis may be different for BA and PA. The results of our CV simulation studies supported that the difference in CV responses is due to faster electron-transfer from PA to the electrochemically generated ^*Br2*^Fc^+^ as compared to that of BA (67 vs 19 s^–1^, respectively–see Figure S1 and Supporting Information for details). Further computational and experimental investigations are necessary to discern the governing factors influencing the electron transfer rates from the two primary benzylic amines to ^*Br2*^Fc^+^ (e.g., lower inner- or outer-sphere reorganization energy or possible inner-sphere electron-transfer via the pyridine moiety).

### Benzylamine Electrocatalytic Oxidation

The utility of ^*Br2*^Fc as the redox mediator for oxidation of BA was then probed under anaerobic controlled-potential electrolysis, see Supporting Information for details.

**Scheme 2:**
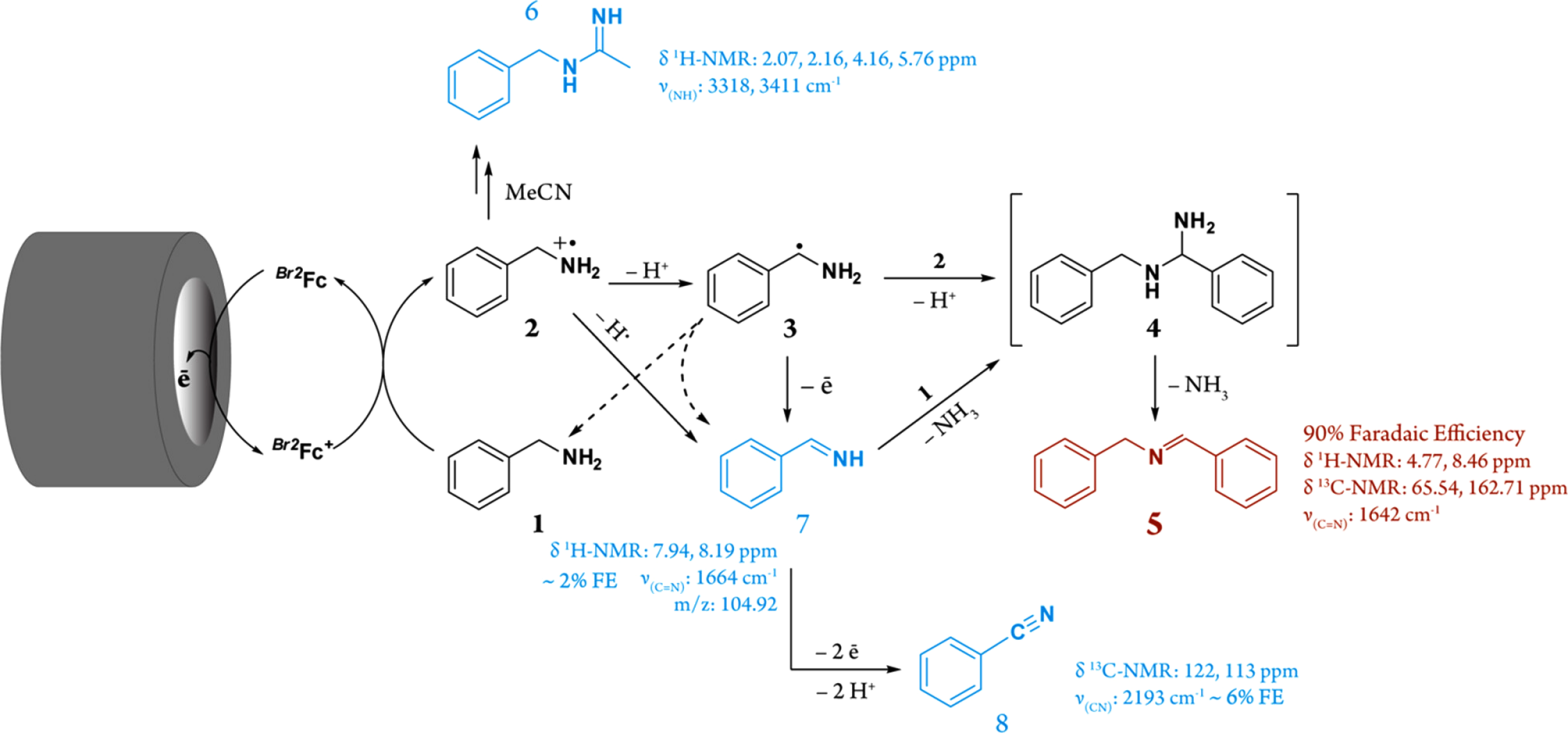
Proposed Mechanism for the Electrocatalytic Oxidation of Benzylamine Mediated by ^*Br2*^Fc The major product is shown in red, and minor products formed during electrocatalysis are displayed in blue.

After purification, we observed the coupled imine product, *N*-(benzylidene)benzylamine (**5**), as the major product of the reaction, with a Faradaic Efficiency of 90% (FE, calculated by eq 1, where *z*, *n*, *F*, and *Q* are the electrons needed to form the product, the number of moles of the product that were obtained, Faraday’s Constant, and the number of coulombs passed during the electrolysis). Our proposed mechanism for this reaction as well as some of the key product characterization details are shown in Scheme 2.
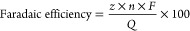
1

Characterization data collected from the anaerobic electrochemical oxidation of benzylamine mediated by ^*Br2*^Fc show the formation of the coupled imine product, typically formed upon the oxidation of benzylamine (Figures S2–S7). As is discussed in several studies of amine oxidation,^40−42^ the first electron oxidation step forms a radical cation on the nitrogen. In our E_r_C_i_′ mechanism, BA (**1**) transfers one electron to the electrochemically generated ^*Br2*^Fc^+^ to regenerate ^*Br2*^Fc in the rate-limiting step. That affords the radical cation (**2**) and results in a dramatic acidification and significant weakening of the α-amino or benzylic C–H bond.^43,44^ This radical cation intermediate can undergo various irreversible chemical reactions. Deprotonation of the amine radical cation at the α-position generates a benzyl radical (**3**) which can couple to an amine radical cation to form an intermediate species (**4**), which loses ammonia to form the coupled product (**5**). Additionally, the benzyl radical can either undergo disproportionation to form the original benzylamine along with an aldimine (**7**), or a one-electron oxidation followed by deprotonation to form the aldimine, which with the addition of benzylamine can form the coupled product (**5**). The transimination of **7** with **1** could also form the couple product (**5**) through intermediate **4**. Alternatively, a direct hydrogen atom transfer (HAT) from the weakened benzylic C–H bond of the amine radical cation followed by a deprotonation can generate the aldimine. Minor side reactions during electrolysis can also include the amine radical cation reacting with acetonitrile/solvent to form an amidine species (**6**).^45,46^ Finally, the aldimine can be further oxidized to form benzonitrile (**8**). Although the latter is a minor side reaction here, this transformation is of particular interest as the amine/nitrile redox systems could be leveraged for the development of hydrogen economy.^47^

It is important to note that hydrolysis of the imine to a benzaldehyde can also occur due to aerobic conditions during purification, however there was no evidence of such products prior to column chromatography.

### 2-Picolylamine Electrocatalytic Oxidation

The electrocatalytic oxidation of PA mediated by ^*Br2*^Fc was performed using the same experimental conditions as that of BA, with minor changes to the procedure (see Supporting Information for details). Based on the characterization data after purification (Figures S8–S11), it was evident that the electrolysis of PA was more significantly impacted by the trace amounts of dioxygen present, as our results showed benzylic oxygenated products.^8,48,49^

**Scheme 3:**
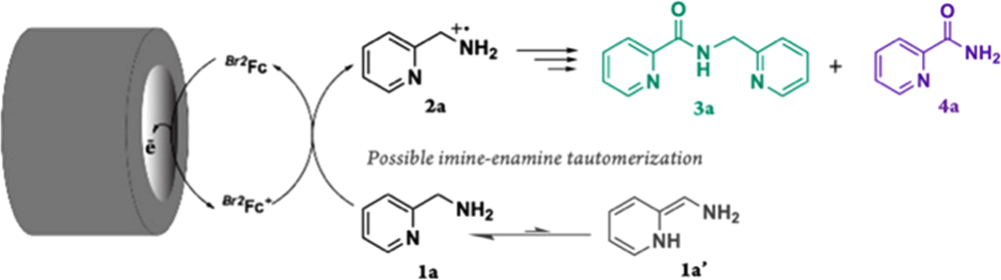
Products Formed as a Result of the Electrocatalytic Oxidation of 2-Picolylamine Mediated by ^*Br2*^Fc The major product is shown in green, and the minor product is displayed in purple.

The products observed are shown in Scheme 3, with the major product being a dimer structure containing an amide (**3a**), and the minor product being picolinamide (**4a**). Surprisingly, the nonoxygenated dimer product with a structure similar to the major product (**5**) of benzylamine oxidation was not observed. While, such a nonoxygenated dimer and/or picolinamide, **4a**,^20,48,50−52^ have been reported as the main products of the aerobic oxidation of PA, **3a** has only been observed when oxygen-atom transfer (OAT) agents, such as sodium hypochlorite (NaOCl)^53^ or a mixture of iodine and *tert*-butyl hydroperoxide,^54^ were used.

Comparing the product profiles of the anaerobic electrolysis of the two substrates points to the significant impact of the heterocycle, i.e., pyridine ring, in PA on the overall reactivity and favoring the oxygenation reaction. This intriguing difference in reactivity between BA and PA could be due to an imine–enamine tautomerization possibly present for PA (1a and 1a’ in Scheme 3), where the enamine tautomer is more susceptible to aerobic oxygenation. A similar imine–enamine tautomerization has been established as a crucial process in the oxidation reaction of benzylic C–H bonds of 2- and 4-benzylpyridine.^8,55^

## Conclusion

Electrochemical oxidation of amines using a redox mediator presents many benefits. Most notably, fouling of the electrode surface is prevented, and the potential at which the catalytic oxidation reaction occurs (E_*cat/2*_) is greatly reduced. Our electrochemical studies reveal that the rate limiting step of the E_r_C_i_′ mechanism, a one-electron transfer between the amine and ^*Br2*^Fc^+^, was faster for 2-picolylamine as compared to benzylamine. Additionally, we have established an anaerobic electrocatalytic oxidation method for the oxidation of benzylamine with a Faradaic efficiency of 90% forming the desired coupled imine product while suppressing an excess of problematic side reactions such as hydrolysis or overoxidation of the substrate that can occur under aerobic conditions or from the use of stoichiometric ferricenium-based oxidants. Further exploration of electrochemical cell design, reaction engineering, and performance optimization such as the use of flow systems and/or porous electrodes are required for developing a practical process and scale-up operations. The reactivity profile of 2-picolylamine significantly differs from that of benzylamine leading to oxygenated products, i.e., amides, possibly due to the presence of an imine–enamine tautomerization, however, it is evident that the system was effective at oxidizing the starting material. Additional computational studies along with chemical means such as anaerobic electrolysis of other picolylamine isomers, are necessary to understand the role of the heterocycle moiety in the reaction mechanisms.

## Experimental Section

### General Methods

All chemicals and solvents were of commercially available grade, unless otherwise noted. Acetonitrile (MeCN) and toluene were purchased from Sigma-Aldrich. All solvents were further purified by passing through a 60 or 18 cm long activation alumina column under argon using a solvent purification system (Innovative Technologies or Inert PureSolve Micro). Acetonitrile was then bubbled with argon for 45–60 min and stored in the glovebox over 3 Å molecular sieves for at least 72 h prior to use.

Infrared (IR) spectra were obtained using a PerkinElmer Spectrum 65 Fourier Transform IR (FT-IR). All NMR spectra were recorded on a JEOL 500 MHz instrument. The chemical shifts were referenced against the CH_3_ (^1^H NMR) and C≡N (^13^C NMR) shifts for the MeCN-*d*_3_ solvent. Electrochemical data was collected using a Bio-Logic SP-200 potentiostat. CV simulations were conducted using the DigiElch software.

Benzylamine (>99.0%) and 1,1′-dibromoferrocene (>98%) were purchased from Tokyo Chemical Industry (TCI). Silver nitrate (>99.9%) and potassium chloride (99%) were purchased from Alfa Aesar, as was the 0.180 mm thick Nafion N-117 membrane. 2-Picolylamine/2-aminomethylpyridine (98%) and tetra-*n*-butylammonium hexafluorophosphate (98%) were purchased from Oakwood Chemicals. Deuterated acetonitrile (MeCN-*d*_3_, 99.8%) was purchased from Cambridge Isotope Laboratories. Distilled water was further purified by a PURELAB flex 1 Analytical Ultrapure Water System (ELGA) for a specific resistance of 18.2 MΩ·cm at 25 °C.

### Electrochemical Measurements

All electrochemical data are plotted according to the polarographic convention. To avoid instabilities in the potentiostat, the *iR* drop was corrected for only 85% of the uncompensated solution resistance during the cyclic voltammetry and bulk electrolysis measurements through positive feedback using the Bio-Logic EC-Lab software.

#### Cyclic Voltammetry

A three-electrode setup was used for all voltammetry experiments with a 3.0 mm glassy carbon disk working electrode (WE; cylindrical, 7.07 mm^2^ surface area), a carbon rod counter electrode (CE), and a leak-free Ag/AgCl reference electrode (RE; LF2 filled with 3.4 M KCl_(aq)_ from Innovative Instruments, Inc.). The reference electrodes were stored in either a 0.05 M H_2_SO_4_ aqueous solution or a saturated KCl aqueous solution between experiments. The potentials were referenced to the Ag/AgCl electrode by first measuring the reduction potential of the ferrocene/ferricenium couple under identical solvent/electrolyte conditions. All experiments were scanned anodically then cathodically at room temperature, in a Vacuum Atmospheres OMNI-Lab inert atmosphere glovebox filled with nitrogen (<0.5 ppm of O_2_ and H_2_O) or in the case of amine containing solutions, the cell was purged with acetonitrile saturated argon. All electrodes were cleaned with acetone and nanopure water before and after use.

#### Bulk Electrolysis

Reactions were performed in a custom-built H-cell (13 mL volume each side) where the glassy carbon plate (60 × 9 × 2 mm; only half of the plate was immersed in the electrolyte) working and Ag/AgNO_3_ (0.01 M in MeCN) reference electrodes were separated from the Pt mesh counter electrode using a 0.180 mm thick Nafion N-117 membrane (Ion Power). See Figures S12 and S13 for images of the bulk electrolysis setup. The “counter solution” was comprised of only the electrolyte, [(*n*Bu)_4_N][PF_6_] (0.1 M) and the “working solution” was comprised of the electrolyte (0.1 M), ^*Br2*^Fc (1 mM), and the substrate of interest (1 M benzylamine or 500 mM 2-picolylamine). The solutions (leaving out the amines) were made in the glovebox and brought out to fill each side of the cell, which was under argon. The substrate was then added to the working solution under argon. Acetonitrile saturated argon was bubbled into the H-cell to ensure that no solvent evaporation would take place during electrolysis. The potential was kept at 0.950 V vs Ag/AgCl, and the reaction continued at room temperature while the “working solution” was stirred using a 10 × 2 mm stir bar at a rate of 800 rpm until the resulting current was unchanging. The experiments lasted approximately 70 h (Q ≈ 37 C for BA) with a Faradaic efficiency of 90% for benzylamine oxidation to **5** and (Q ≈ 15.5 C for PA) with a Faradaic efficiency of 60% for 2-picolylamine oxidation to **3a**.

### Purification and Characterization of the Products

#### Benzylamine Oxidation

The first fraction collected from column chromatography, mostly the coupled product **5** (approximately 33.5 mg), was collected as a pale-yellow oil and characterized. FT-IR (cm^–1^): ν_(C=N)_ = 1642. ^1^H NMR (MeCN-*d*_*3*_, 500 MHz; δ, ppm): 4.77 (d, 2H), 8.46 (s, 1H), 7.27 (q, 1H), 7.35 (d, 4H), 7.45 (m, 3H), 7.78 (dd, 2H). ^13^C NMR (MeCN-*d*_*3*_, 500 MHz, δ, ppm): 65.54 (C–N), 127.83, 128.96 (d), 129.40, 129.65, 131.67, 137.46, 140.86, 162.71 (C=N).

The second fraction contained a mixture of compounds (∼7 mg) as shown by the NMR spectra, including an amidine **6** (∼5 mg, redox neutral) and nitrile **8 (<**1 mg, FE ≈ 6%). The evidence for the amidine is as follows: FT-IR (cm^–1^): ν_(N–H)_ = 3318, 3411 (sh), 1598; ν_(C=N)_ = 1665. ^1^H NMR (MeCN-*d*_*3*_, 500 MHz; δ, ppm): 2.07 (s, 3H), 4.16 (d, 2H), 5.76 (br. s, 1H). ^13^C NMR (MeCN-*d*_*3*_, 500 MHz; δ, ppm): 19.80 (CH_3_), 47.38 (CH_2_). Evidence for the nitrile is as follows: FT-IR (cm^–1^): ν_(C≡N)_ = 2194; ^13^C NMR (MeCN-*d*_*3*_, 500 MHz; δ, ppm): 122.44.

#### 2-Picolylamine Oxidation

The second fraction collected from column chromatography, picolinamide **4a**, was a pale-yellow oil and characterized **(∼**2.2 mg, FE ≈ 22%). ^1^H NMR (MeCN-*d*_*3*_, 500 MHz; δ, ppm): 6.13 (br. s, 1H), 7.76 (br. d, 1H), 7.53 (t, 1H), 7.92 (t, 1H), 8.09 (d, 1H), 8.59 (d, 1H). ^13^C NMR (MeCN-*d*_*3*_, 500 MHz, δ, ppm): 122.82, 127.42, 138.44, 149.44, 150.89, 164.58 (C=O).

The third fraction collected was determined to be the coupled product **3a** and was characterized **(**∼10 mg, FE ≈ 60%). ^1^H NMR (MeCN-*d*_*3*_, 500 MHz; δ, ppm): 4.70 (d, 2H), 7.25 (td, 1H), 7.34 (d, 1H), 7.54 (td, 1H), 7.73 (td, 1H), 7.94 (td, 1H), 8.12 (d, 1H), 8.55 (d, 1H), 8.63 (d, 1H), 8.95 (br. s, 1H). ^13^C NMR (MeCN-*d*_*3*_, 500 MHz; δ, ppm): 45.04 (CH_2_), 122.32, 122.74, 123.16, 127.41, 137.68, 138.57, 149.46, 149.97, 150.87, 158.42, 165.09 (C=O).

## Supporting Information Available

Details on the characterization of major and minor products of the oxidation reactions, as well as cyclic voltammetry data (PDF)
