# Supplementary material for: Electrocatalytic Anaerobic Oxidation of Benzylic Amines Enabled by Ferrocene-Based Redox Mediators
Source: Organometallics. 2024 Aug 11;43(20):2557–64. doi: 10.1021/acs.organomet.4c00219 (PMC11523463; doi:10.1021/acs.organomet.4c00219)
Supplement: Supplementary file 1 — om4c00219_si_001.pdf [file om4c00219_si_001.pdf]

Supporting Information for

Electrocatalytic Anaerobic Oxidation of Benzylic Amines Enabled by Ferrocene-Based Redox Mediators

Amy L. Waldbusser and Shabnam Hematian*

Department of Chemistry and Biochemistry, University of North Carolina at Greensboro, Greensboro, NC 27402, United States
* Email: s_hemati@uncg.edu

Contents:

1. Cyclic voltammetry data including E_{cat} and $E_{cat/2}$ values & CV simulations
2. Characterization data for the major and minor products of electrocatalytic benzylamine oxidation mediated by ^{Br2}Fc
3. Characterization data for the major and minor products of electrocatalytic 2-picolylamine oxidation mediated by ^{Br2}Fc
4. Images of the bulk electrolysis setup

1. Cyclic voltammetry data

a. E_{cat} and $E_{cat/2}$ values

Table S1. E_{cat} and $E_{cat/2}$ values for the direct and redox mediated oxidation of benzylamine and 2-picolylamine in MeCN with 100 mM of $[(n\text{Bu})_4\text{N}][\text{PF}_6]$ as the supporting electrolyte.

	Direct oxidation		^{Br2}Fc mediated oxidation	
	E_{cat}	$E_{cat/2}$	E_{cat}	$E_{cat/2}$
Benzylamine	1.55	1.229	0.950	0.798
2-Picolylamine	1.62	1.234	0.950	0.789

b. CV simulations

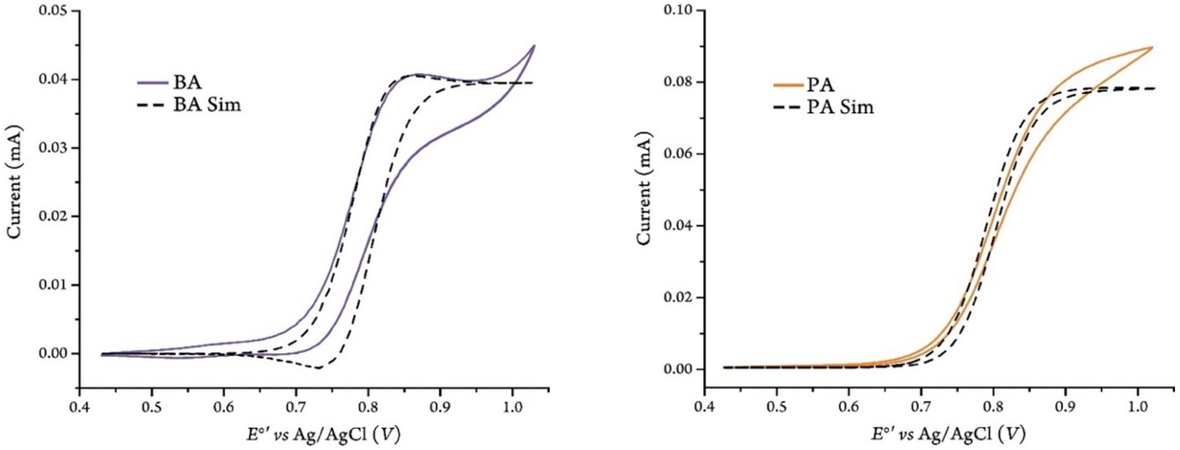

Figure S1. Experimental (solid) and simulated (black dashed) cyclic voltammograms of 1 mM ^{Br2}Fc in the presence of 100 mM BA (purple, left) or 100 mM PA (orange, right) at 100 mVs^{-1} for an E_rC_i' mechanism. Simulation parameters: T = 298.2 K, electrode area = 0.0707 cm^2, α = 0.5, k_s = 1 cm s^{-1}, $E°_{Br2Fc}$ = 0.763 V, D_{Br2Fc} = 1.81×10^{-5} cm^2 s^{-1}, D_{Br2Fc^+} = 1.50×10^{-5} cm^2 s^{-1}, R_u = 65 Ohm. The heterogeneous electron transfer constant between ^{Br2}Fc and the electrode was arbitrarily assumed to be 1 cm s^{-1} as it would be identical for both electrochemical systems. The obtained rate constant of the electron transfer from the electrochemically generated ^{Br2}Fc$^+$ to the amine, k_e, was estimated to be 19 and 67 s^{-1} for the BA and PA oxidation, respectively.

2. Characterization data for the major and minor products of electrocatalytic benzylamine oxidation mediated by ^{Br2}Fc

Anaerobic controlled-potential electrolysis was performed in a separated "H-cell" in order to separate the processes at the working electrode from those at the counter. The ^{1}H-NMR spectra of the crude solution after electrolysis showed that some BA remained in the mixture, and there was another compound present whose shifts matched those expected of the coupled product **5**. The solvent, along with some of the benzylamine, was removed from the "working electrode" solution under vacuum. A minimal amount of toluene was added to the dark brown sludge and the mixture was filtered to separate the products from the electrolyte. The solvent was again removed under vacuum. This mixture consisted of approximately 83% of unreacted BA while ~17% were the final products including the coupled product **5** plus the minor species (**6**, **7**, and **8**). Thin layer chromatography on alumina with a 60% hexanes/40% ethyl acetate mixture revealed two major fractions, besides benzylamine, which were separated through column chromatography of the same conditions. Through column chromatography, we were able to separate the benzylamine from two other species present in the solution, as BA was no longer seen in the NMR spectra of the mixture after the column. The first fraction (~33.5 mg) to elute from the column was the major coupled product plus a minimal amount of benzaldehyde which possibly formed from hydrolysis of **7** during purification (FE ≈ 2%). The second fraction (7.0 mg isolated) contained a mixture of minor products (**6** and **8** with the latter having FE of ~6%). Following is the characterization data of both fractions.

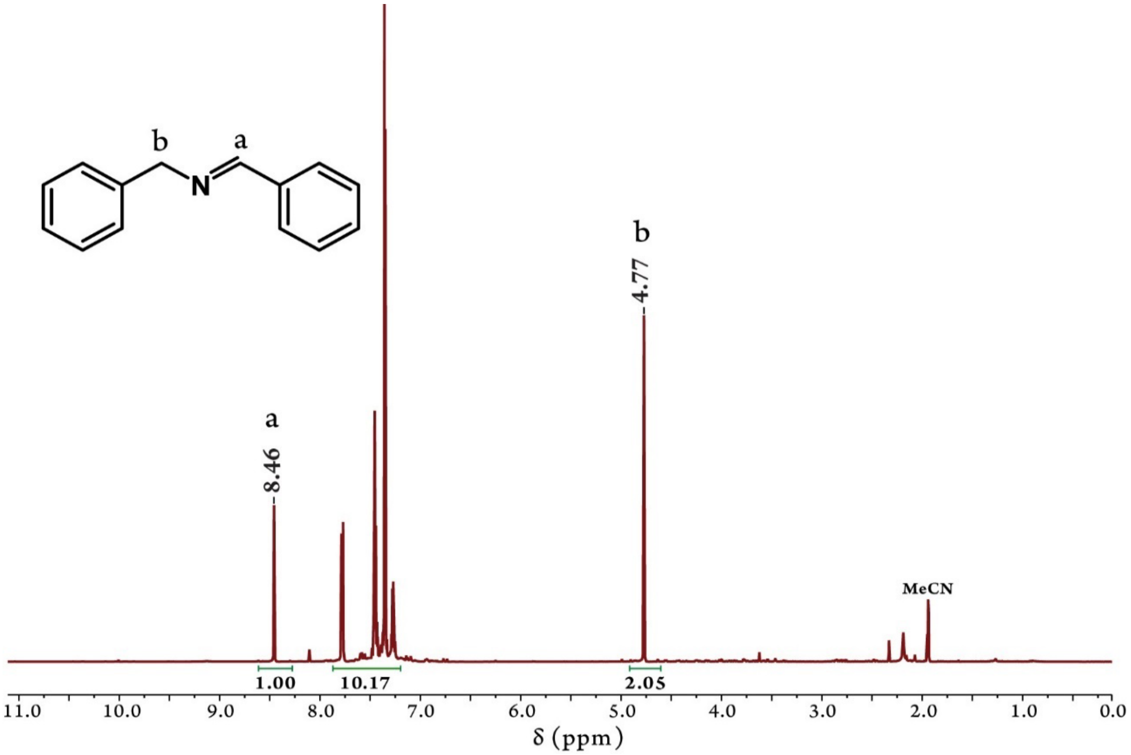

Figure S2. The ^{1}H-NMR spectra of the coupled product (**5**) formed as a result of the controlled-potential electrolysis of benzylamine using ^{Br2}Fc as a redox mediator, collected in acetonitrile-d_3 at room temperature.

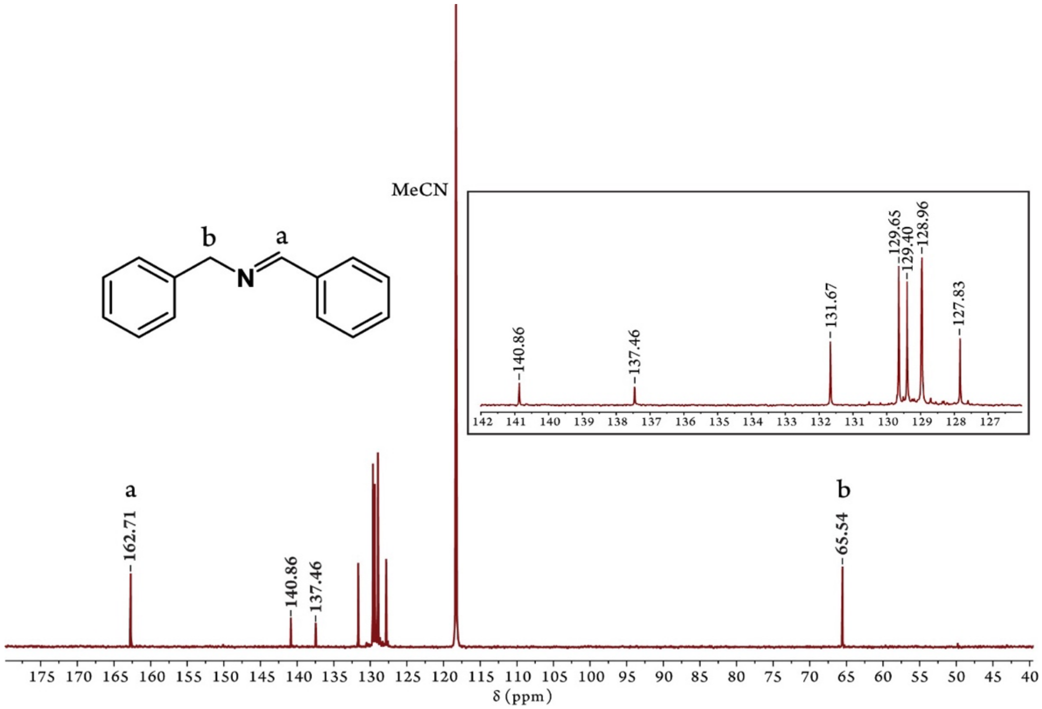

Figure S3. The ^{13}C-NMR spectra of the coupled product (**5**) formed as a result of the controlled-potential electrolysis of benzylamine using ^{Br2}Fc as a redox mediator, collected in acetonitrile-d_3 at room temperature. Inset shows the aromatic region.

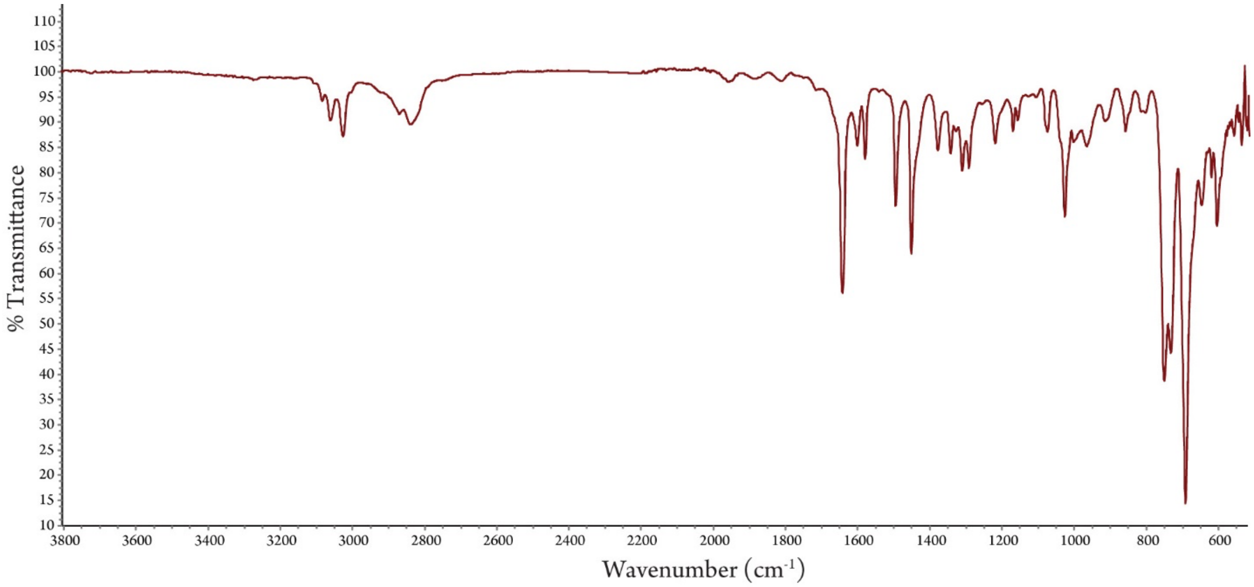

Figure S4. IR Spectrum of the major product of benzylamine oxidation (**5**). The peak at 1642 cm^{-1} corresponds to the C=N stretching mode.

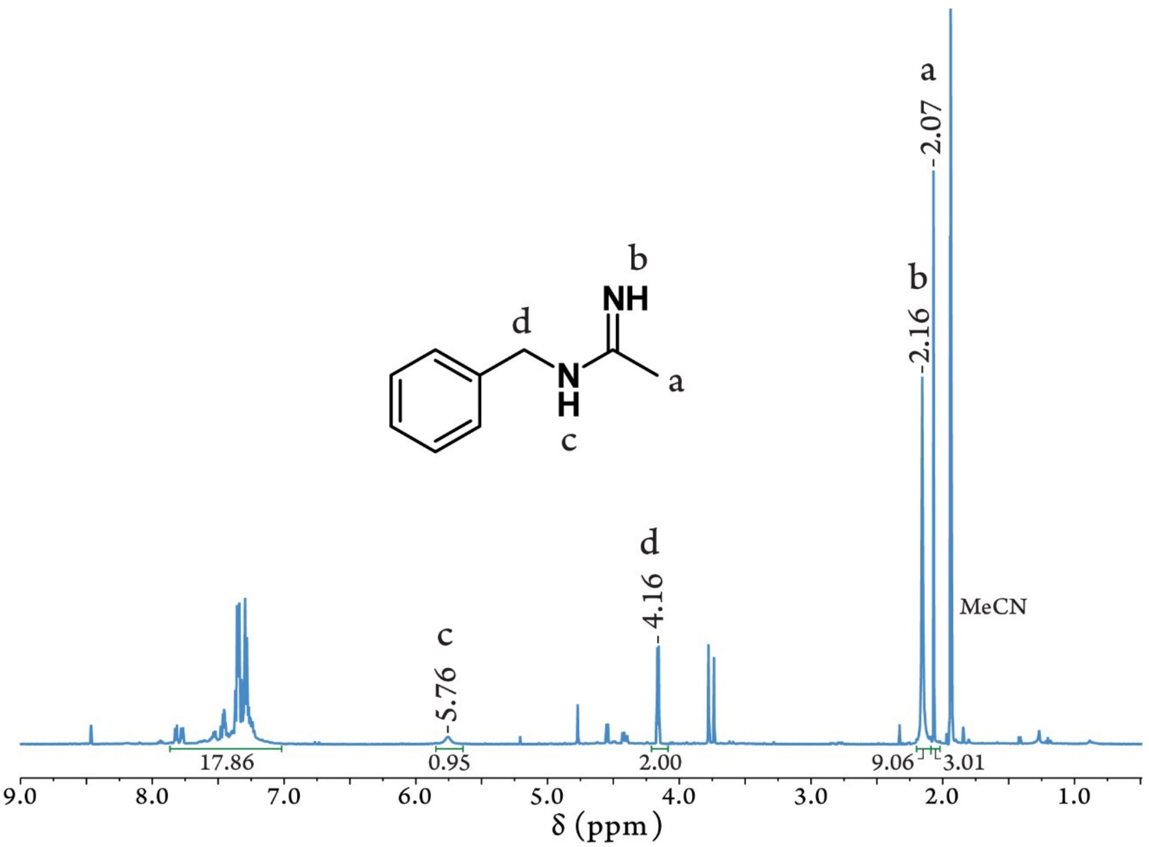

Figure S5. The ^{1}H-NMR spectra of the minor species mixture obtained from the controlled-potential electrolysis of benzylamine using ^{Br2}Fc as a redox mediator, with emphasis on peaks that correspond to a benzyl-amidine structure (**6**). Collected in acetonitrile-d_3 at room temperature.

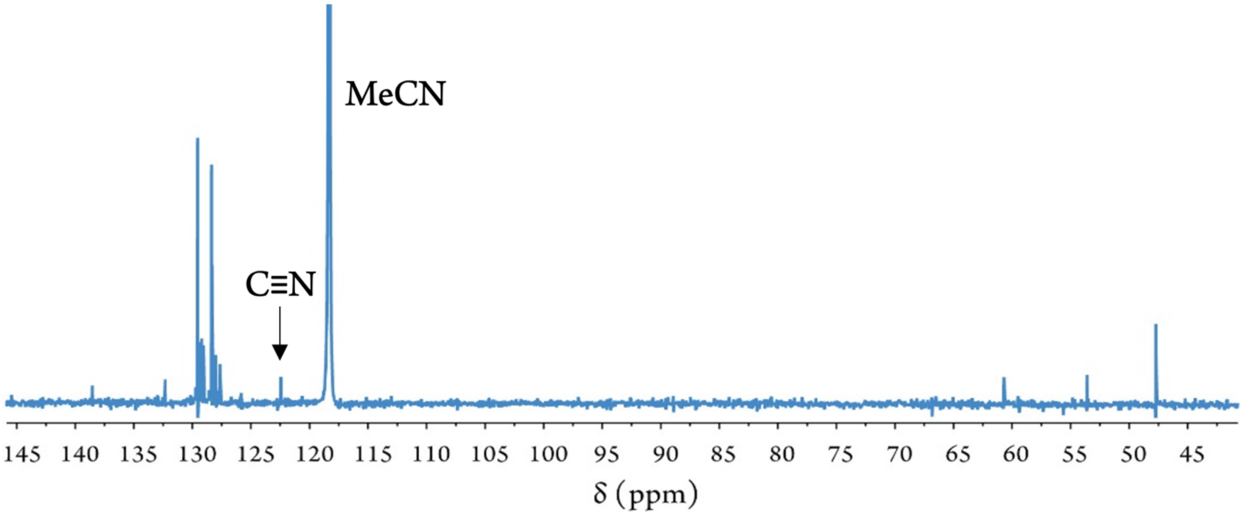

Figure S6. ^{13}C-NMR spectrum of the minor products of benzylamine oxidation, collected in acetonitrile-d_3 at room temperature.

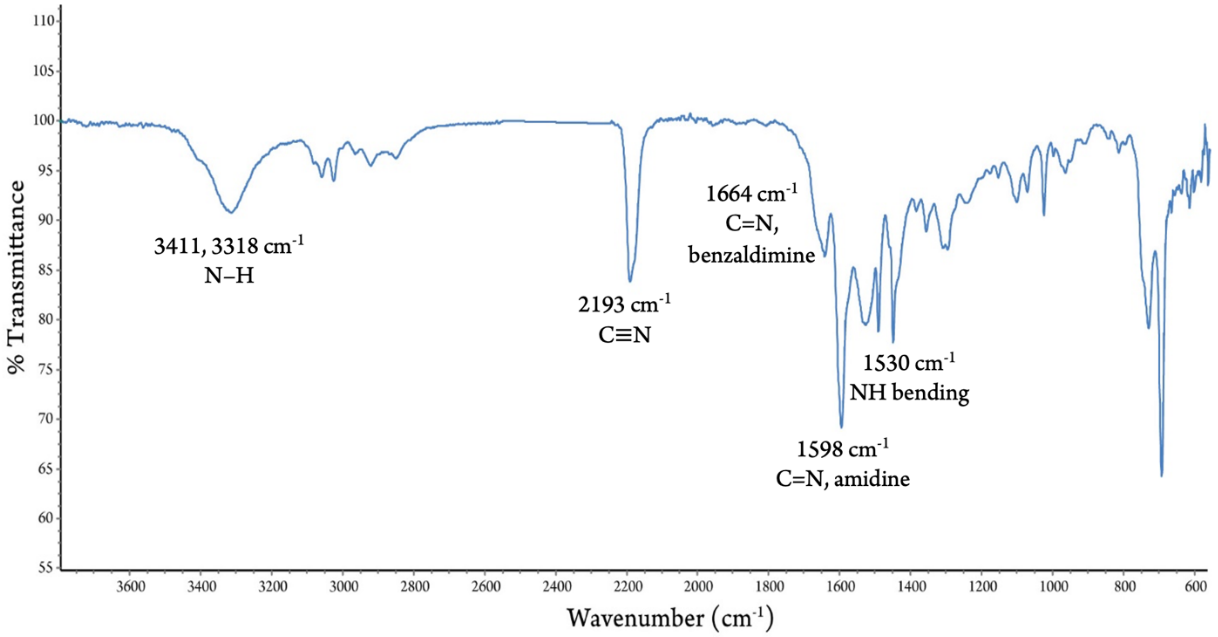

Figure S7. IR spectrum of the minor products (**6**, **7**, and **8**) of benzylamine oxidation.

3. Characterization data for the major and minor products of electrocatalytic 2-picolylamine oxidation mediated by ^{Br2}Fc

This experiment was performed in the same manner as benzylamine oxidation, with exceptions being that 500 mM of PA was used instead of 1 M, and the column conditions were slightly more polar than those used for benzylamine. Here, a 60% ethyl acetate and 40% hexanes mixture eluted five fractions, two of which were considered to be the major (**3a**) and minor (**4a**)products due to the amount purified. Following is the characterization of those purified products. Specific aromatic assignments were possible through 2D-NMR spectroscopy (COSY, NOESY, HSQC – not shown). Approximate isolated amounts of the products **3a** and **4a** were ~10.0 mg (FE ≈ 60%) and ~2.2 mg (FE ≈ 22%), respectively. The purification and identification of other products which formed during the PA electrolysis were unsuccessful.

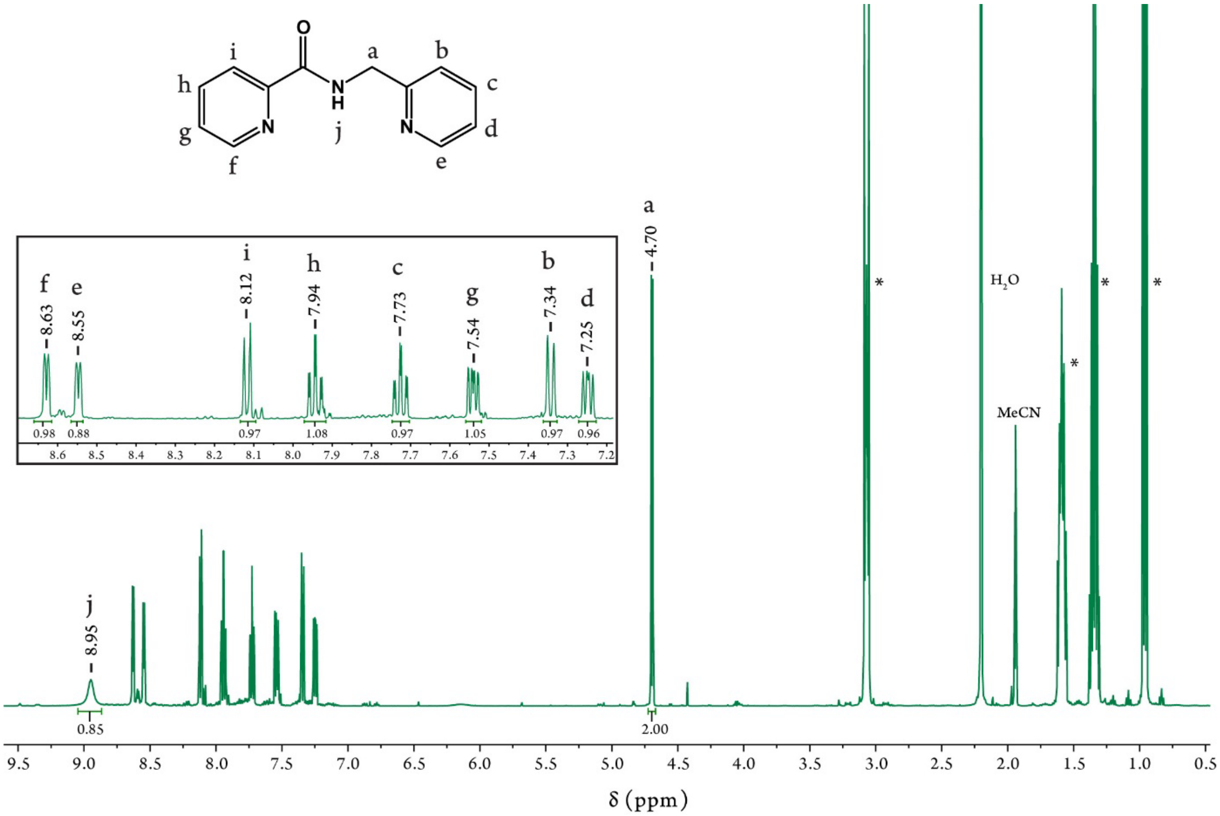

Figure S8. The ^{1}H-NMR spectra of the major product (**3a**) formed as a result of the controlled-potential electrolysis of 2-picolylamine using ^{Br2}Fc as a redox mediator, collected in acetonitrile-d_3 at room temperature. The * symbol corresponds to peaks from the electrolyte, $[(n\text{Bu})_4\text{N}][\text{PF}_6]$.

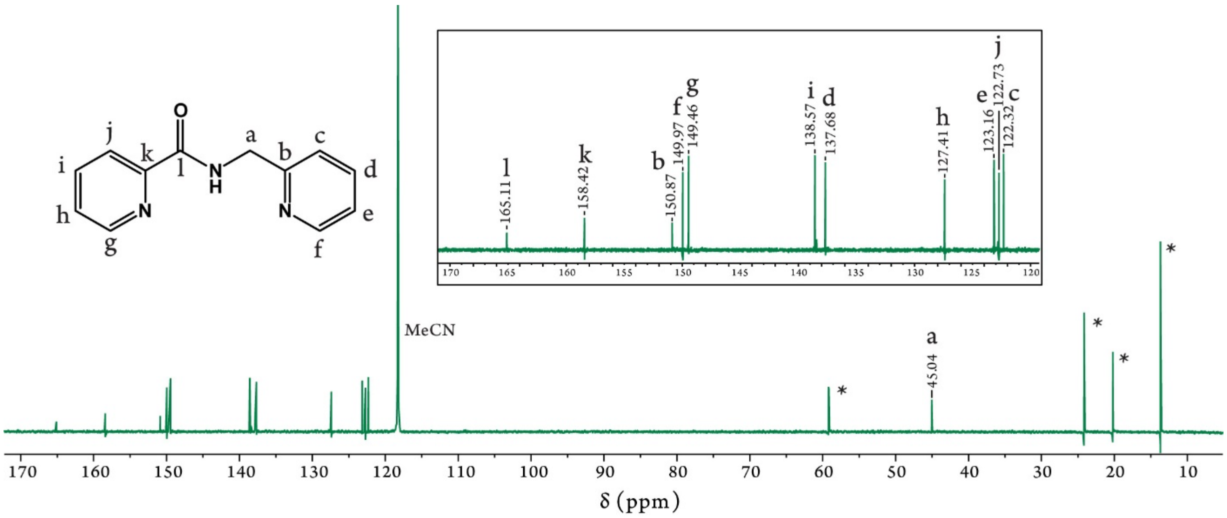

Figure S9. The ^{13}C-NMR spectra of the major product (**3a**) formed as a result of the controlled-potential electrolysis of 2-picolylamine using ^{Br2}Fc as a redox mediator, collected in acetonitrile-d_3 at room temperature. Inset shows the aromatic region. The * symbol corresponds to peaks from the electrolyte, $[(n\text{Bu})_4\text{N}][\text{PF}_6]$.

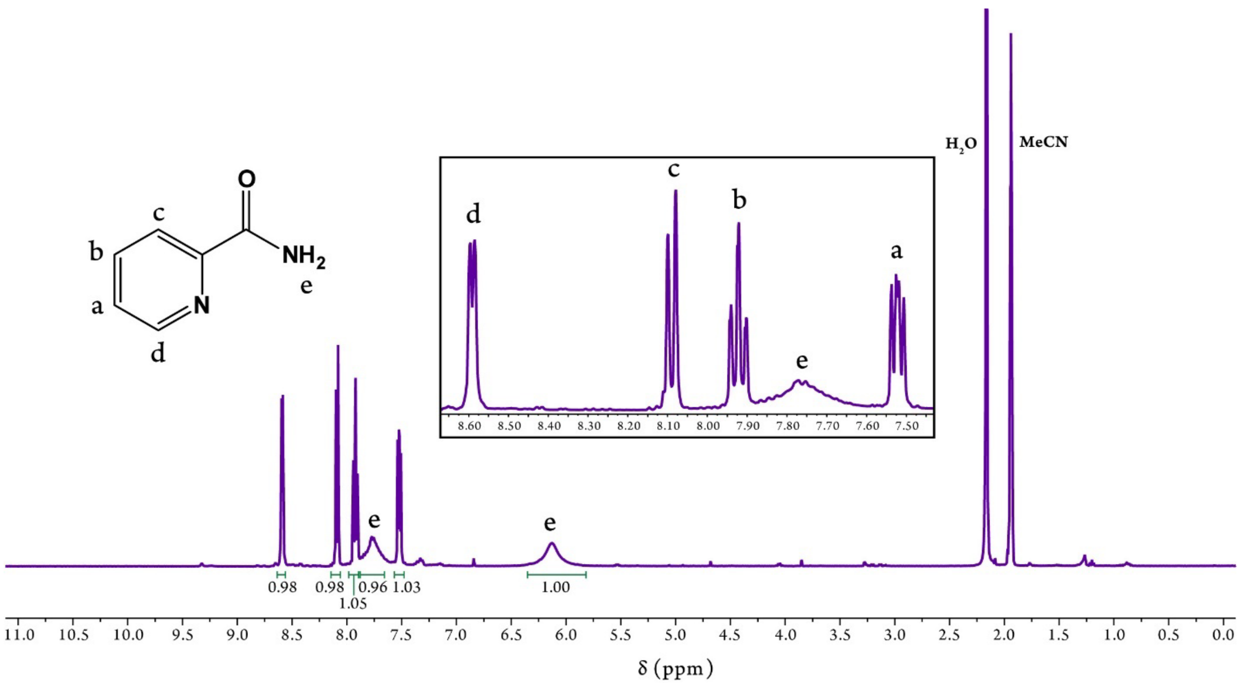

Figure S10. The ^{1}H-NMR spectra of the minor product (**4a**) formed as a result of the controlled-potential electrolysis of 2-picolylamine using ^{Br2}Fc as a redox mediator, collected in acetonitrile-d_3 at room temperature. Inset shows the aromatic region.

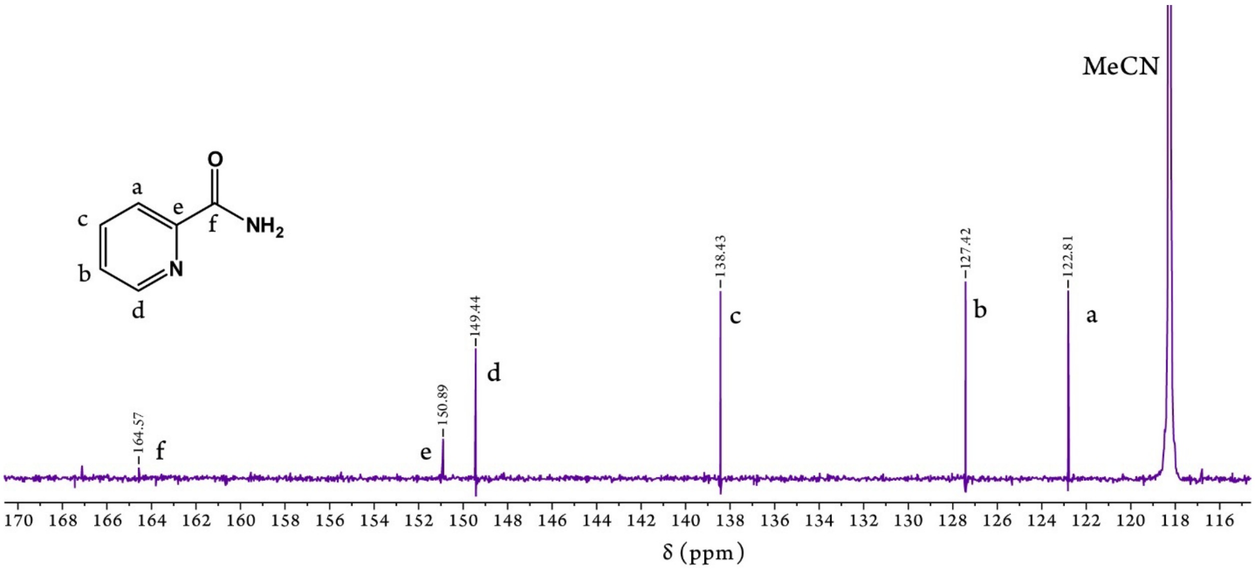

Figure S11. The ^{13}C-NMR spectra of the minor product (**4a**) formed as a result of the controlled-potential electrolysis of 2-picolylamine using ^{Br2}Fc as a redox mediator, collected in acetonitrile-d_3 at room temperature.

4. Images of the bulk electrolysis setup

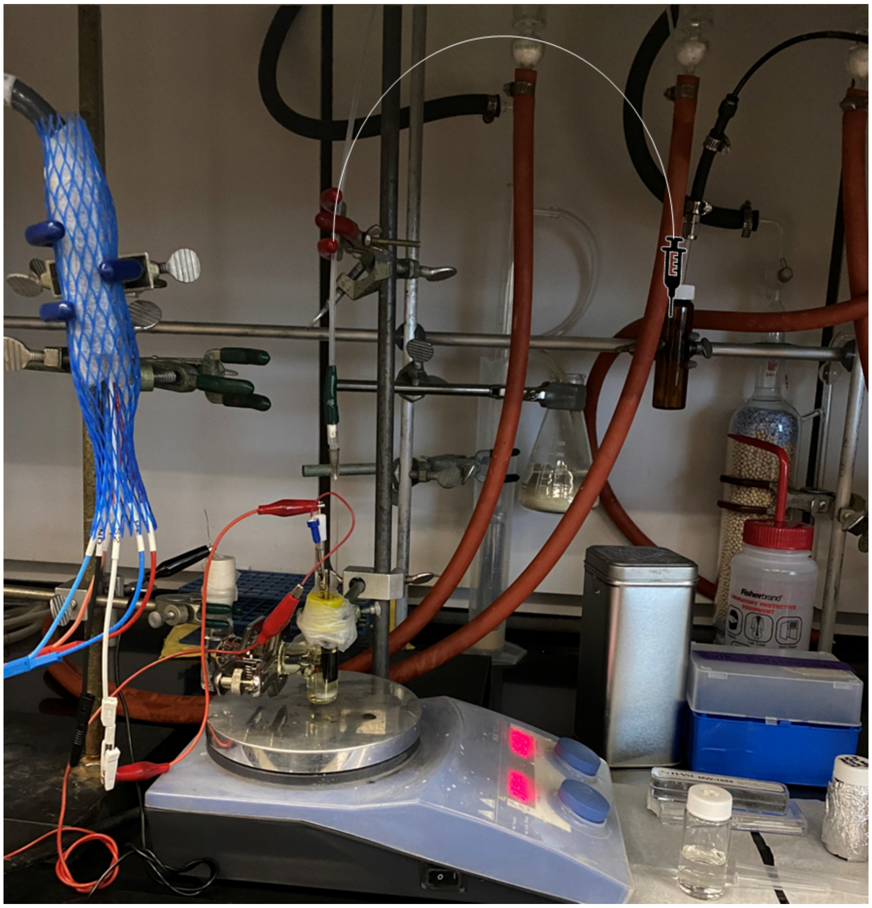

Figure S12. Our setup of the bulk electrolysis experiments, with an image of a needle inserted to highlight that the acetonitrile-saturated argon was being bubbled into the working solution from the brown vial.

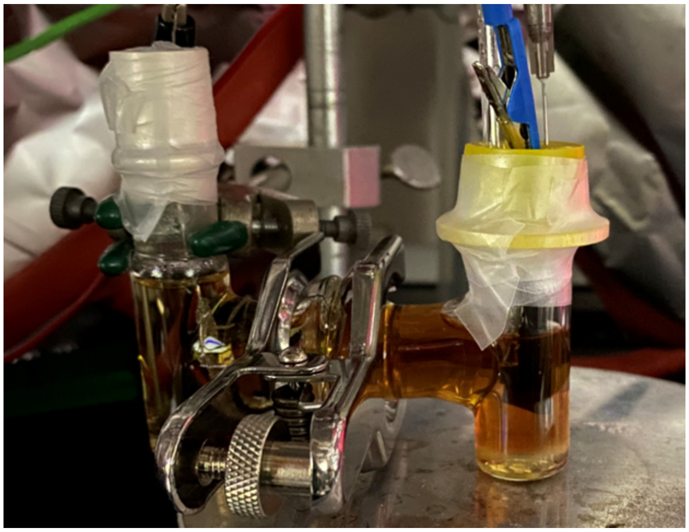

Figure S13. A closer image of the H-cell setup used in the bulk electrolysis experiments.
